# LAMA2 Congenital Muscle Dystrophy: A Novel Pathogenic Mutation in Bulgarian Patient

**DOI:** 10.1155/2018/3028145

**Published:** 2018-07-25

**Authors:** Ivanka Dimova, Ivo Kremensky

**Affiliations:** Laboratory of Genomic Diagnostics, Center of Molecular Medicine, Department of Medical Chemistry and Biochemistry, Medical University Sofia, Zdrave Str. 2, 1431 Sofia, Bulgaria

## Abstract

Congenital muscle dystrophies (CMD) are genetically and clinically heterogeneous hereditary myopathies mainly with autosomal recessive type of inheritance. The most common form worldwide is considered to be merosin-deficient muscle dystrophy type 1A, called MDC1A (due to laminin-*α*2 defects as a result of* LAMA2* gene mutation), accounting for 30-40% of total cases of CMD. The exact molecular and clinical diagnoses, respectively, are a prerequisite for the most effective treatment; sometimes orphan drugs exist for some rare diseases. One of such drugs is Tarix, which was FDA approved and announced in 2016 for treatment of MDC1A. Here we present a patient diagnosed postmortem as having early-onset* LAMA2*-related muscular dystrophy as a result of mutations in* LAMA2, * identified by Sanger sequencing in his parents: a novel nonsense mutation c.4452T>A in exon 31, identified in the mother, and a known pathogenic nonsense mutation c.2901C>A in exon 21, detected in the father. The truncating nature of both nonsense mutations made the clinical presentation severe and the outcome fatal. Genetic analysis in such cases of muscle dystrophy is of utmost impact, because it makes the correct diagnosis with at least some specific options for treatment, makes the prognosis depending on the severity of mutation discovered, determines reproductive risk, and offers prophylaxis in the family by means of prenatal or preimplantation diagnostics.

## 1. Introduction

Congenital muscle dystrophies (CMD) are genetically and clinically heterogeneous hereditary myopathies mainly with autosomal recessive type of inheritance. They are characterized by muscle hypotonia from the birth or early infancy, disturbed physical development, static or progressive muscle weakness, joint contractures, and dystrophic changes in histological examination of muscles. In some forms there is also involvement of central nervous system with different degree of mental retardation and seizures. Some children die in infancy, whereas others can live into adulthood with only minimal disability [1].

The genetic defects affect different proteins with essential roles in muscle development and function: defects of structural proteins (laminin-*α*2, collagen, integrin, and plectin), defects of glycosylation (Walker-Warburg syndrome, muscle-eye brain disease, and Fukuyama CMD), proteins of the endoplasmic reticulum and nucleus (selenoprotein, selenocysteine insertion sequence-binding protein 2, laminin A/C), and mitochondrial membrane protein (choline kinase beta). Only about 25-50% of patients with CMD have an identifiable genetic mutation.

Different incidence and mortality rate have been reported in the literature depending on the type of CMD and population studies. The average frequency of CMD is 1-9 per 100 000, ranging from 1 per 125 000 in Italy to 1 per 16 000 in Sweden. The most common form worldwide is considered to be merosin-deficient muscle dystrophy type 1A, called MDC1A (due to laminin-*α*2 defects), accounting for 30-40% of total cases of CMD, although in some geographical regions other forms are prevalent, such as Fukuyama CMD in Japan and muscle-eye-brain (MEB) disease in Finland.

A diagnosis of CMD is made based upon a thorough clinical evaluation, a detailed patient history, identification of characteristic symptoms, and a variety of specialized tests including biopsy of affected muscle tissue that may reveal characteristic changes to muscle fibers, electromyography, specialized blood tests, immunohistochemistry, magnetic resonance imaging (MRI), and molecular genetic testing.

Genetic testing is of paramount important since it makes the precise diagnosis, determines the affected protein/process, and allows making prognosis depending on the severity of mutation discovered. The exact molecular and clinical diagnoses, respectively, are a prerequisite for the most effective treatment; sometimes orphan drugs exist for some rare diseases. Such drugs are Tarix, which was FDA approved and announced in 2016 for treatment of MDC1A, and Omigapil for either Ulrich or MDC1A subtypes of CMD.

## 2. Case Presentation

We report here a boy, born after 1st normal pregnancy by a normal mechanism with weight of 3200 g; the neonatal period was normal. At time of birth some deformations were noticed in wrists, knees, and ankles, together with decreased muscle tonus. At age of 3 months there was no active movement and control of the head and there was poor support of the legs. No tendon and bone reflexes were established. Laboratory investigation, performed at age of 3 months, showed highly increased levels of the enzymes ASAT (149 IU/l), GGT (121 IU/l), and LDH (1260 IU/l); high levels of creatine kinase CPK were detected (4309 U/l). Together with clinical symptoms this constellation indicated a disorder from the group of congenital myopathies. Electromyography detected spontaneous activity from fibrillations and prolonged insertion activity. The reported potentials had decreased duration and amplitude. Interferential type with low amplitude in relatively poor muscle contraction was registered. The clinicians concluded that it corresponds to primary muscle disorder. Transfontanel echography showed mild enlargement of lateral ventricles. The child was directed to physiotherapist. At that time (1999) molecular genetic testing was not routinely introduced in our country and DNA analysis for suspected genes was not done.

At the age of 1.5 years, the patient had muscle hypotonia, contracture in the foot, and limited extension in the knee. He actively moved the hands and sat after putting him stable, but he did not stand up, stay up, and walk. There was areflexia in the lower extremities. The creatin kinase (CK) was highly increased (2072 IU/l).

At 4 years, the following dysmorphism was noted: triangle face, high forehead, open mouth, low positioned asymmetric ears, and dolichocephalism. The muscle and subcutaneous fat tissues were highly reduced. There were contractures in all major joints and sunken chest.

In the next years the child has been often hospitalized because of infections, febrility, and intoxication. At 10 years, during the course of bilateral pneumonia, acute respiratory deficiency was developed. The patient died with pulmonary and brain edema and total cachexia. There was histological examination after death with the following observations: muscle dystrophy and atrophy in all skeletal muscles including diaphragm, representing different size of myocytes, fibrous tissues among the cells, and diffuse lipomatosis.

The congenital muscular dystrophies (CMD) are heterogeneous group of autosomal recessive disorders presenting in infancy with muscle weakness, contractures, and dystrophic changes on skeletal-muscle biopsy. Approximately 40% of patients with CMD have a primary deficiency (MDC1A) of the laminin alpha-2 chain of merosin (laminin-2) due to autosomal recessive variants in the LAMA2 gene.

Sequencing of the entire coding region (exons 1-65) and all intron-exon boundaries of the LAMA2 gene was performed in father and mother of the deceased child after obtaining an informed consent. The reference sequence was according to Genbank accession number Z26653, with the A of the ATG start codon on position 1.

Two genetic variants were identified in the mother: a pathogenic variant and a variant of unknown significance.

*(1) A Heterozygous LAMA2:c.4452T>A Variant in Exon 31 of the LAMA2 Gene*. This substitution is a nonsense variant predicted to lead to the alteration of a cysteine into a premature stop codon on position 1484 (LAMA2:p.Cys1484X). The LAMA2:c.4452T>A variant may result in a truncated LAMA2 protein or diminished LAMA2 mRNA due to mRNA decay. The LAMA2:c.4452T>A variant is a novel variant not previously described in other patients or in controls. Due to its truncating nature, it is classified as a pathogenic variant according to the MutaDATABASE criteria (http://www.MutaDATABASE.org).

*(2) A Heterozygous LAMA2:c.3494G>C Variant in Exon 24 of the LAMA2 Gene*. This substitution is a missense variant predicted to lead to the substitution of a serine by a threonine on amino acid position 1165 of the resulting LAMA2 protein (LAMA2:p.Ser1165Thr). The LAMA2:c.3494G>C variant is a novel variant not previously described in other patients or in controls. Therefore, it is classified as a variant of unknown significance according to the MutaDATABASE criteria (http://www.MutaDATABASE.org). In silico prediction programs were as follows: Polyphen-2: benign; SIFT: tolerated; Mutation Taster: polymorphism.

In the father of the deceased child a pathogenic variant was identified.

*(i) A Heterozygous LAMA2: c.2901C>A Variant in Exon 21 of the LAMA2 Gene*. This substitution is a nonsense variant predicted to lead to the alteration of a cysteine into a premature stop codon on position 967 (LAMA2:p.Cys967X). The LAMA2:c.2901C>A variant may result in a truncated LAMA2 protein or diminished LAMA2 mRNA due to mRNA decay. The LAMA2:c.2901C>A variant is a known variant previously described in other patients. Due to its truncating nature, it is classified as a pathogenic variant according to the MutaDATABASE criteria (http://www.MutaDATABASE.org).

## 3. Discussion

*LAMA2*-related muscular dystrophy is due to defect in the gene encoding the *α*2 subunit, which together with *β*1 and *γ*1 chains form laminin-211 (Lm-211), the most prevalent laminin found in basement membrane (BM), surrounding muscle fibers, and also in the Schwann cells of peripheral nerves [2]. Other components of BM during embryonic development are Lm-411 (*α*4, *β*1, and *γ*1) and Lm-511 (*α*5, *β*1, and *γ*1). The therapeutic strategy in LAMA2 dystrophy aims at improving BM stability. The introduction of additional proteins to BM by transgenic expression ameliorated muscle structure and function and opened promise for suffering patients [3]. First experiments started in LAMA2-deficient mouse with forced expression of* Lama1*, encoding laminin-*α*1, by gene therapy vectors, but therapeutic efficiency was poor based on the large insert size of* LAMA1. *Then new strategy was presented by using linker proteins (agrin, laminin-*α*1, and nidogen-1) that can be incorporated into adeno-associated virus (AAV) vectors. However, systemic delivery of AAV has not been reported for muscular dystrophy patients [4].

Loosing structural stability, the contracting muscle fibers start to degenerate in LAMA2 MD and trigger a cascade of secondary events, such as apoptosis/necrosis, inflammation, and fibrosis. These pathogenic mechanisms assumed the use of some inhibitors as treatment options in patients with muscular dystrophy. For example, the angiotensin II type 1 receptor blocker losartan inhibits fibrosis and improves the disease in mouse models for Duchenne muscular dystrophy and* LAMA2* MD [5–7]. Recently orphan drug Tarix (TXA127), which counteracts the classical renin angiotensin system, was announced and approved by FDA for LAMA2 MD, Marfan Syndrome, and amyotrophic lateral sclerosis (ALS).

Merosin-deficient congenital muscular dystrophy 1A (MDC1A) resulting from mutations in the LAMA2 gene was found as the most common among all congenital muscle dystrophies, in 37.4% of 249 unrelated individuals referred to neurogenetics services [8]. The subclassification of the disease is based on the degree of merosin expression, genotype, and clinical features. Compared to the residual merosin group, patients with absent merosin had an earlier presentation, were more likely to lack independent ambulation, or require enteral feeding and ventilatory support [9]. Patients with residual merosin and later presentation often carried missense, splice site mutation, and less frequently frameshift mutations [10].

The case, presented in the manuscript, is an illustration of our approach when there is no DNA sample from the affected child and we base the assumptions only on the clinical documentation. This is a difficult situation in the practice of genetic counseling and step-by-step genetic protocol is applied, starting with the most suspected genetic diagnosis. The patient was diagnosed postmortem as having early-onset* LAMA2*-related muscular dystrophy based on the results of mutations in* LAMA2* (including a novel nonsense mutation: c.4452T>A/p.Cys1484X) identified by Sanger sequencing in his parents. The truncating nature of both nonsense mutations made the clinical presentation severe and the outcome fatal. Genetic analysis in such cases of muscle dystrophy is of utmost impact, because it makes the correct diagnosis with at least some specific options for treatment, makes the prognosis, determines reproductive risk, and offers prophylaxis in the family by means of prenatal or preimplantation diagnostics.

## References

[1] Mah J. K., Korngut L., Fiest K. M. (2016). A systematic review and meta-analysis on the epidemiology of the muscular dystrophies. *Canadian Journal of Neurological Sciences*.

[2] Reinhard J. R., Lin S., McKee K. K. (2017). Linker proteins restore basement membrane and correct LAMA2-related muscular dystrophy in mice. *Science Translational Medicine*.

[3] McKee K. K., Crosson S. C., Meinen S., Reinhard J. R., Rüegg M. A., Yurchenco P. D. (2017). Chimeric protein repair of laminin polymerization ameliorates muscular dystrophy phenotype. *The Journal of Clinical Investigation*.

[4] Yurchenco P. D., McKee K. K., Reinhard J. R., Rüegg M. A. (2017). Laminin-deficient muscular dystrophy: Molecular pathogenesis and structural repair strategies. *Matrix Biology*.

[5] Cohn R. D., van Erp C., Habashi J. P. (2007). Angiotensin II type 1 receptor blockade attenuates TGF-ß-induced failure of muscle regeneration in multiple myopathic states. *Nature Medicine*.

[6] Habashi J. P., Judge D. P., Holm T. M. (2006). Losartan, an AT1 antagonist, prevents aortic aneurysm in a mouse model of Marfan syndrome. *Science*.

[7] Elbaz M., Yanay N., Aga-Mizrachi S. (2012). Losartan, a therapeutic candidate in congenital muscular dystrophy: Studies in the dy 2J/dy 2J mouse. *Annals of Neurology*.

[8] Sframeli M., Sarkozy A., Bertoli M. (2017). Congenital muscular dystrophies in the UK population: Clinical and molecular spectrum of a large cohort diagnosed over a 12-year period. *Neuromuscular Disorders*.

[9] Geranmayeh F., Clement E., Feng L. H. (2010). Genotype-phenotype correlation in a large population of muscular dystrophy patients with LAMA2 mutations. *Neuromuscular Disorders*.

[10] Marques J., Duarte S. T., Costa S. (2014). Atypical phenotype in two patients with LAMA2 mutations. *Neuromuscular Disorders*.

